# Tail-Suspension Model of Simulated Microgravity-Induced Functional Dyspepsia in Rats: Behavioral, Motility, and Brain–Gut Peptide Alterations

**DOI:** 10.3390/ijms27114915

**Published:** 2026-05-29

**Authors:** Wei Li, Yang Li, Fengzhong Wang, Hengrui Qi, Bei Fan, Guangyou Wang, Qiong Wang

**Affiliations:** 1 Institute of Food Science and Technology, Chinese Academy of Agricultural Sciences, Beijing 100193, China; 15230231316@163.com (W.L.); engsut1@gmail.com (Y.L.); wangfengzhong@caas.cn (F.W.); qrhengruiqi0329@163.com (H.Q.); fanbei517@163.com (B.F.); 2 Neurobiology Laboratory, Harbin Medical University, Harbin 150081, China; 3 National Nanfan Research Institute (Sanya), Chinese Academy of Agricultural Sciences, Sanya 572024, China

**Keywords:** tail-suspension model, functional dyspepsia, brain–gut axis, simulated microgravity, translational research

## Abstract

Animal models are essential for elucidating human disease mechanisms and advancing translational research. Here, we used a well-established rat tail-suspension model to investigate the pathophysiological changes associated with simulated microgravity-induced functional dyspepsia (FD) and to evaluate its utility for preclinical to clinical translation. Thirty male Wistar rats were randomly assigned to control, simulated weightlessness using hindlimb unloading (HU), and domperidone groups. The HU model was induced by 21-day tail suspension, a widely accepted ground-based platform for simulating microgravity. Behavioral tests (sucrose preference, novelty-suppressed feeding), gastrointestinal motility measurements (gastric emptying, intestinal propulsion), and serum brain–gut peptide levels were assessed. Gastric and hypothalamic gene expression was analyzed by qRT-PCR. The model successfully recapitulated key FD phenotypes, including anxiety/depression-like behaviors, reduced gastric emptying and intestinal propulsion, and systemic brain–gut peptide imbalance—characterized by decreased excitatory peptides [substance P (SP), gastrin (GAS), motilin (MTL), ghrelin] and increased inhibitory peptides [vasoactive intestinal peptide (VIP), cholecystokinin (CCK), calcitonin gene-related peptide (CGRP), nesfatin-1] in serum. Consistent transcriptional dysregulation was observed in gastric and hypothalamic tissues. Hippocampal brain-derived neurotrophic factor (BDNF) was decreased, and colon 5-hydroxytryptamine (5-HT) increased, with no organic gastric lesions. Domperidone treatment significantly ameliorated behavioral abnormalities and gastrointestinal dysmotility, partially reversed brain–gut peptide imbalances at both protein and transcriptional levels, and restored hippocampal BDNF. These findings demonstrate that the rat tail-suspension model provides a reproducible platform for studying microgravity-induced FD, implicating brain–gut axis dysregulation. Domperidone’s therapeutic effects highlight the model’s utility for evaluating countermeasures against spaceflight-associated digestive dysfunction.

## 1. Introduction

Animal models play a critical role in elucidating the complex pathophysiology of human diseases and enabling the translation of ground-based findings into clinical countermeasures. During long-term spaceflight, the microgravity environment exerts multifaceted effects on the astronauts’ digestive system, including delayed gastric emptying, potential alterations in intestinal barrier function, and gut microbiota dysbiosis [1,2,3]. These physiological changes can lead to a cluster of dyspeptic symptoms in astronauts, such as postprandial fullness, early satiety, and upper abdominal pain, affecting their nutritional status, physical and mental health, and posing challenges to the execution of long-duration missions. The pathogenesis of functional dyspepsia (FD) is complex and is considered a disorder of brain–gut interaction involving multiple factors [4]. Its core pathophysiological basis includes gastrointestinal motility disorders (e.g., delayed gastric emptying) and visceral hypersensitivity [5]. Concurrently, psychosocial factors (e.g., stress) can participate in the development and progression of FD through the bidirectional regulation of the brain–gut axis. Studies have confirmed that the microgravity environment itself can induce increased negative emotions such as anxiety and depression in both astronauts and experimental animals [6,7,8], suggesting that the weightless environment may exacerbate digestive tract dysfunction by affecting central emotional states and subsequently acting via the brain–gut axis.

The brain–gut axis, as a bidirectional communication system between the central nervous system and the gastrointestinal tract, relies on various core molecules for signal transduction, among which brain–gut peptides are considered key regulators [9]. These peptides, distributed in both the brain and the gut, such as vasoactive intestinal peptide (VIP) [10], substance P (SP) [11], and ghrelin [12], collectively participate in regulating gastrointestinal motility, secretion, perception, and even feeding behavior. Research has confirmed that these gut-derived peptide signals can influence brain function through multiple pathways, playing an important role in the regulation of emotion and behavior, thereby closely linking the state of gastrointestinal function with psychological emotions [13].

In ground-based simulation studies, the tail suspension method is often used to establish rat models of weightlessness [14]. This model effectively simulates several key physiological changes induced by the space microgravity environment, including muscle atrophy, bone loss, and digestive system dysfunction, and is a classic method for studying the physiological effects of weightlessness [15].

Based on this model, further studies have found that simulated weightlessness significantly affects gastrointestinal motility and may interfere with the normal expression and function of key brain–gut peptides such as ghrelin and substance P (SP). These changes are consistent with some of the pathological features of functional dyspepsia (FD).

Existing research mostly focuses on phenomenological observation. There is still a lack of systematic research on the detailed molecular and neural circuit mechanisms by which simulated microgravity specifically regulates the brain–gut peptide network to initiate and maintain the FD state. Furthermore, the translational potential of this model, particularly its utility for bridging preclinical findings with clinical applications, remains underexplored. This study characterizes the association between simulated weightlessness, brain–gut axis dysregulation, and FD using a standardized tail-suspension model, emphasizing phenotypic characterization and translational relevance for astronaut health protection.

## 2. Results

### 2.1. Changes in General Condition

Compared with the control group, rats in the model group gradually showed mental fatigue, significantly reduced activity, and fluffy, dull, and lusterless fur approximately one week after suspension, with some rats exhibiting loose stools. Rats in the control group maintained good mental status, responsive behavior, and smooth, shiny fur. Rats in the domperidone group showed improved mental status and increased activity compared with the model group after approximately one week of administration.

Body weight monitoring (Table 1) showed no significant differences among the three groups at the beginning of the experiment (*p* > 0.05). Compared with the control group, body weight gain in the model group was already slower on day 7, and body weight was significantly lower than that of the control group on days 14 and 21 (all *p* < 0.05). Body weight in the domperidone group was significantly higher than that in the model group at the corresponding time points on days 14 and 21 (*p* < 0.05). Changes in food intake were generally consistent with the body weight trend. Food intake in the model group was significantly lower than that in the control group on days 14 and 21 (*p* < 0.05), while the domperidone group showed a trend toward increased food intake compared with the model group, although the differences did not reach statistical significance at some time points.

### 2.2. Behavioral Assessment Results

#### 2.2.1. Sucrose Preference Test

**Figure 1:**
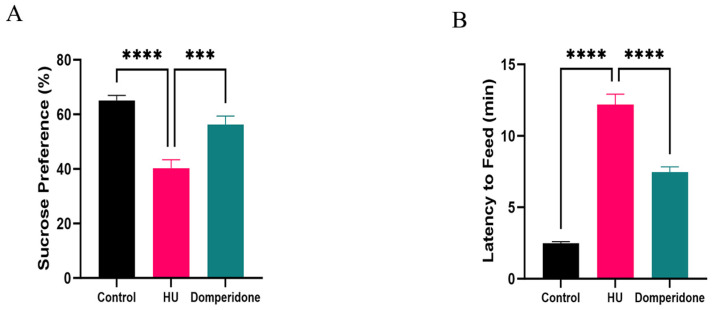
Effects of simulated weightlessness on emotional behavior in rats and the intervention effect of domperidone. (**A**) Sucrose preference percentage of rats in each group, as shown by the sucrose preference test. (**B**) Feeding latency of rats in each group as shown by the novelty-suppressed feeding test. n = 9–10. Control: control group; HU: simulated weightlessness model group; domperidone: domperidone group. *** *p* < 0.001, **** *p* < 0.0001, one-way ANOVA with Tukey’s HSD post hoc test.

Compared with the control group, the sucrose preference percentage of rats in the model group was significantly decreased. Compared with the model group, the sucrose preference percentage of rats in the domperidone group was significantly increased (Figure 1A). Reduced sucrose preference is consistent with a depressive-like state, though it may also reflect reduced food intake or malaise.

#### 2.2.2. Novelty-Suppressed Feeding Test

Compared with the control group, the latency to first feeding in a novel environment was significantly prolonged in rats of the model group. Compared with the model group, the feeding latency of rats in the domperidone group was significantly shortened (Figure 1B). Prolonged feeding latency in this test is commonly associated with anxiety-like behavior; however, it can also reflect reduced appetite, early satiety, or abdominal discomfort—all of which are known to occur in functional dyspepsia. Thus, the observed increase in feeding latency should be interpreted as a composite indicator of altered motivational or emotional state, rather than pure anxiety.

Collectively, these behavioral alterations indicate that simulated weightlessness leads to changes in reward responsiveness and feeding behavior under novel stress, but the specific contributions of emotional disturbance versus dyspeptic symptoms (e.g., postprandial discomfort, early satiety) remain to be disentangled in future studies.

### 2.3. Gastrointestinal Motility Measurement Results

**Figure 2:**
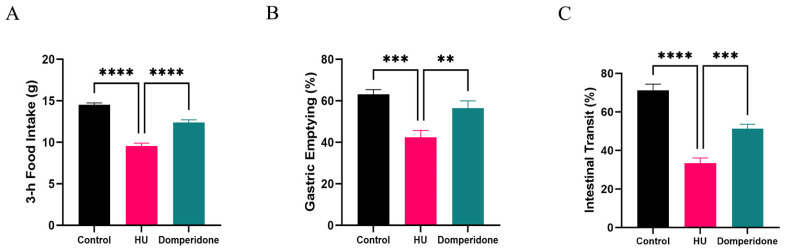
Effects of simulated weightlessness on gastrointestinal function in rats and the intervention effect of domperidone. (**A**) Three-hour food intake; (**B**) gastric emptying rate; (**C**) small intestinal propulsion rate. n = 9–10. Control: control group; HU: simulated weightlessness model group; domperidone: domperidone group. ** *p* < 0.05, *** *p* < 0.001, **** *p* < 0.0001, one-way ANOVA with Tukey’s HSD post hoc test.

To comprehensively evaluate the early effects of simulated weightlessness on feeding behavior and gastrointestinal emptying function, we measured the 3 h food intake after fasting, gastric emptying rate, and small intestinal propulsion rate in rats. The results showed that the 3 h food intake, gastric emptying rate, and small intestinal propulsion rate in the model group were significantly lower than those in the control group. Following domperidone intervention, all three parameters were significantly restored compared with the model group. These results suggest that simulated weightlessness not only inhibits gastrointestinal mechanical emptying but may also further aggravate dyspeptic symptoms by affecting appetite and early satiety, and domperidone exerts improving effects on both aspects (Figure 2).

### 2.4. Gastric Histopathological Results

**Figure 3:**
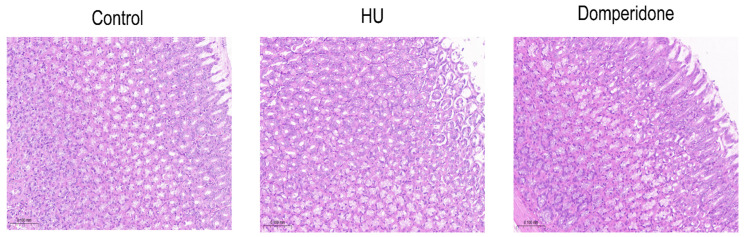
Histopathological observation of rat gastric tissue (HE staining, ×400). The gastric mucosa of rats in all three groups showed intact structure, with well-arranged epithelium and glands. No organic lesions such as inflammatory infiltration, erosion, ulceration, or atrophy were observed.

Histopathological observation of gastric tissue (HE staining) showed that (Figure 3) the gastric mucosal structure was intact in rats of the control group, model group, and domperidone group, with no significant pathological changes observed. Specifically, the gastric mucosal epithelial cells were neatly arranged, the glandular structure was clear and tightly packed, and the cells within the lamina propria exhibited normal morphology. No organic lesions such as inflammatory cell infiltration, erosion, ulceration, or glandular atrophy were detected. These findings further support that this study successfully established a functional dyspepsia model rather than an organic disease model.

### 2.5. Molecular Assay Results

#### 2.5.1. Levels of Brain–Gut Peptides and Neuroactive Factors

To elucidate the potential molecular mechanisms by which simulated weightlessness affects FD from the peripheral circulation and tissue levels, we systematically measured the levels of relevant brain–gut peptides and neuroactive factors using ELISA.

**Figure 4:**
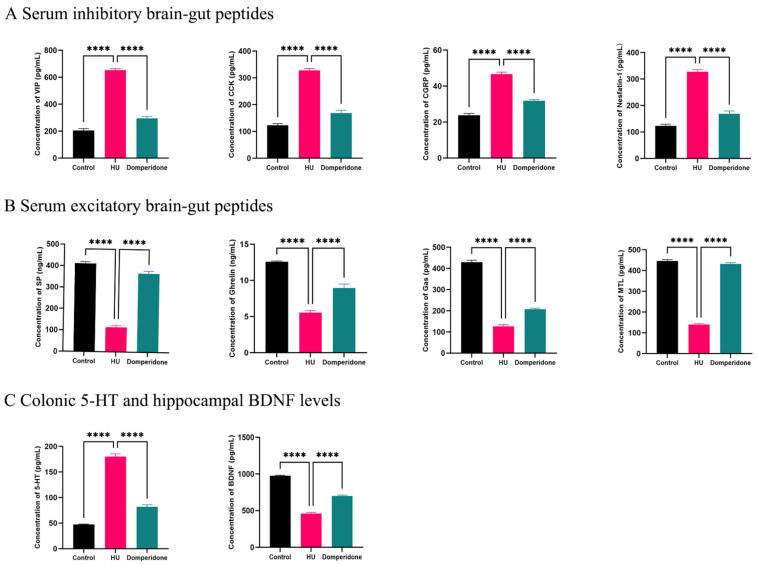
Effects of simulated weightlessness on levels of brain–gut peptides and key factors in rat serum and tissues, and the intervention effect of domperidone. (**A**) Serum levels of brain–gut peptides that inhibit gastrointestinal motility: vasoactive intestinal peptide (VIP), cholecystokinin (CCK), calcitonin gene-related peptide (CGRP), and nesfatin-1. (**B**) Serum levels of brain–gut peptides that promote gastrointestinal motility: substance P (SP), ghrelin, gastrin (GAS), and motilin (MTL). (**C**) Levels of key factors in colon and hippocampal tissues: colon 5-hydroxytryptamine (5-HT) and hippocampal brain-derived neurotrophic factor (BDNF). Control: control group; HU: simulated weightlessness model group; domperidone: domperidone group. **** *p* < 0.0001, one-way ANOVA with Tukey’s HSD post hoc test.

At the serum level, compared with the control group, rats in the model group exhibited a significant imbalance of brain–gut peptides (Figure 4). Specifically, the serum concentrations of brain–gut peptides that inhibit gastrointestinal motility, including vasoactive intestinal peptide (VIP), cholecystokinin (CCK), calcitonin gene-related peptide (CGRP), and the feeding inhibitory factor nesfatin-1, were significantly increased (Figure 4A). Conversely, the concentrations of brain–gut peptides that promote gastrointestinal motility, such as substance P (SP), ghrelin, gastrin (GAS), and motilin (MTL), were significantly decreased (Figure 4B).

Concurrently, abnormal changes in key factors were also observed in local tissues. The level of the excitatory neurotransmitter 5-hydroxytryptamine (5-HT) in the colon tissue of model group rats was significantly increased, while the level of the neurotrophic factor—brain-derived neurotrophic factor (BDNF)—in the hippocampal tissue was significantly decreased (Figure 4C).

Domperidone intervention effectively reversed these abnormal changes. Compared with the model group, the serum levels of VIP, CCK, CGRP, and nesfatin-1, as well as the colon 5-HT level, were significantly decreased in the domperidone group; meanwhile, the serum levels of SP, ghrelin, GAS, and MTL, as well as the hippocampal BDNF level, were significantly restored (Figure 4A–C). These results indicate that simulated weightlessness leads to widespread dysregulation of key signaling molecules from the periphery to the central nervous system, and that domperidone exerts multi-target regulatory effects on these abnormalities.

#### 2.5.2. Gastric and Hypothalamic mRNA Expression of Brain–Gut Peptides and Their Receptors

**Figure 5:**
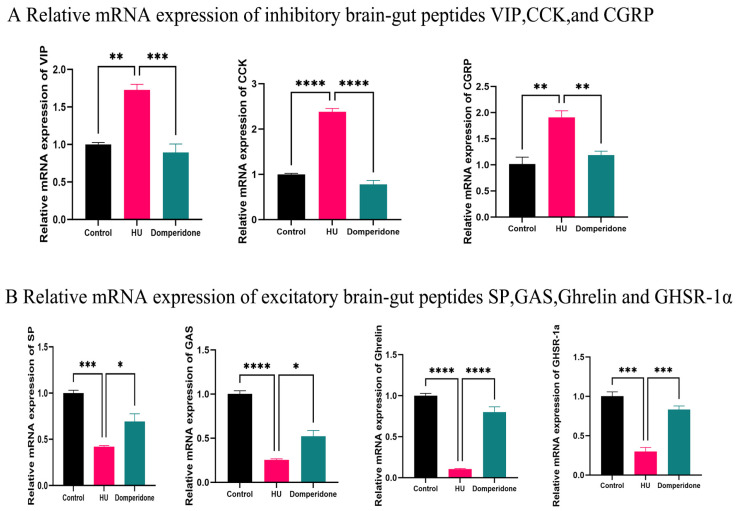
Effects of simulated weightlessness on mRNA expression of brain–gut peptides in rat gastric tissue and the intervention effect of domperidone. (**A**) mRNA expression of inhibitory brain–gut peptides *VIP*, *CCK*, and *CGRP*; (**B**) mRNA expression of excitatory brain–gut peptides *SP*, *GAS*, and the ghrelin system (*ghrelin*, *GHSR-1a*). Control: control group; HU: simulated weightlessness model group; domperidone: domperidone group. * *p* < 0.05, ** *p* < 0.01, *** *p* < 0.001, **** *p* < 0.0001, one-way ANOVA with Tukey’s HSD post hoc test.

To investigate the molecular regulatory mechanisms underlying the effects of simulated weightlessness on FD, we examined the mRNA expression of key brain–gut peptides and their receptors at the transcriptional level in gastric tissue (peripheral effector organ) and hypothalamic tissue (central regulatory hub) (Figure 5).

In gastric tissue, compared with the control group, the mRNA expression of genes encoding brain–gut peptides that inhibit gastrointestinal motility—*VIP*, *CCK*, and *CGRP*—was significantly upregulated in the model group (all *p* < 0.01) (Figure 5A). Conversely, the mRNA expression of genes encoding brain–gut peptides that promote gastrointestinal motility—*SP* and *GAS*—as well as genes of the ghrelin system, including *ghrelin* and its receptor growth hormone secretagogue receptor 1a (*GHSR-1a*), was significantly downregulated (Figure 5B). Following domperidone treatment, the mRNA expression of *VIP*, *CCK*, and *CGRP* in gastric tissue was significantly downregulated, while the mRNA expression of *ghrelin* and *GHSR-1a* was significantly upregulated (Figure 5A,B).

**Figure 6:**
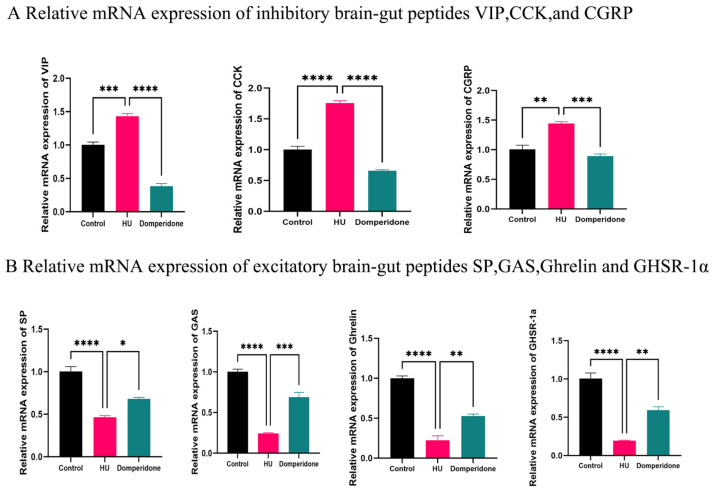
Effects of simulated weightlessness on mRNA expression of brain–gut peptides in rat hypothalamic tissue and the intervention effect of domperidone. (**A**) mRNA expression of inhibitory brain–gut peptides *VIP*, *CCK*, and *CGRP*; (**B**) mRNA expression of excitatory brain–gut peptides *SP*, *GAS*, and the ghrelin system (*ghrelin*, *GHSR-1a*). Control: control group; HU: simulated weightlessness model group; domperidone: domperidone group. * *p* < 0.05, ** *p* < 0.01, *** *p* < 0.001, **** *p* < 0.0001, one-way ANOVA with Tukey’s HSD post hoc test.

In hypothalamic tissue, rats in the model group similarly exhibited a comparable pattern of transcriptional dysregulation. Compared with the control group, the mRNA expression of *VIP*, *CCK*, and *CGRP* in the hypothalamus was significantly increased (Figure 6A), while the mRNA expression of *SP*, *GAS*, *ghrelin*, and *GHSR-1a* was significantly decreased (Figure 6B). Following domperidone intervention, the mRNA expression of *VIP*, *CCK* and *CGRP* in the hypothalamus was significantly suppressed, whereas the mRNA expression of ghrelin and *GHSR-1a* was significantly elevated (Figure 6A,B).

## 3. Discussion

This study systematically reveals that simulated microgravity induces FD-like changes in rats via brain–gut axis dysregulation. Using a 21-day tail-suspension model, we show that simulated weightlessness causes gastrointestinal dysmotility, anxiety/depression-like behaviors, and a widespread imbalance of brain–gut peptides: decreased excitatory peptides (SP, GAS, MTL, ghrelin) and increased inhibitory peptides (VIP, CCK, CGRP, nesfatin-1) in serum, with consistent transcriptional changes in gastric and hypothalamic tissues. Domperidone ameliorates these abnormalities and partially reverses the peptide imbalance. These findings support the tail-suspension model as a valuable preclinical tool for space medicine research.

### 3.1. Brain–Gut Peptide Imbalance: The Core Molecular Mechanism of Weightlessness-Induced FD

The most important finding of this study is the revelation of the widespread imbalance of serum brain–gut peptides under simulated weightlessness. In the model group, serum levels of brain–gut peptides that promote gastrointestinal motility (SP, GAS, MTL, ghrelin) were significantly decreased, while those that inhibit gastrointestinal motility (VIP, CCK, CGRP, nesfatin-1) were significantly increased. This peptide environment, characterized by “inhibition-enhanced and excitation-weakened”, provides a direct molecular basis for explaining the low gastrointestinal motility caused by simulated weightlessness. Among these, the decrease in ghrelin and the increase in VIP are particularly critical. Ghrelin not only potently promotes gastric motility and appetite but also possesses anxiolytic and antidepressant effects [16]. VIP, on the other hand, is a potent relaxant of gastrointestinal smooth muscle, and its overexpression can inhibit gastrointestinal contractions [17]. The opposite directions of change in these two peptides synergistically lead to suppressed gastrointestinal peristalsis and negative emotional changes.

Notably, this study confirmed for the first time at the transcriptional level that the mRNA expression of ghrelin and its receptor GHSR-1a was downregulated, while VIP mRNA expression was upregulated in both gastric tissue and the hypothalamus. This suggests that the regulation of brain–gut peptides by weightlessness occurs at the transcriptional level and involves dual changes in both peripheral and central components. This aligns with recent research progress on the bidirectional regulatory mechanism of the brain–gut axis—the newly proposed “enterolimbic axis” concept emphasizes that gut-derived signals directly affect emotional regulatory centers via the vagus nerve–brainstem–limbic circuitry, while the central state can, in turn, regulate gastrointestinal function [18]. The consistent changes in brain–gut peptides in both central and peripheral components observed in this study provide experimental support for this theoretical framework.

Furthermore, the increase in CCK, a satiety signal peptide and anxiety regulator [19], and nesfatin-1, a novel anorexia- and anxiety-related peptide [20], may jointly exacerbate the anorexia and anxiety-like behaviors in model animals. It is worth noting that the colonic 5-HT level was significantly increased in the model group, which is related to the complex role of 5-HT in the regulation of gastrointestinal motility. 5-HT can both promote intestinal peristalsis and participate in the formation of visceral hypersensitivity [21]; its increase reflects compensatory changes in the local enteric nervous system or activation of inflammation-related signals under weightlessness.

### 3.2. Association Between Emotional Behavioral Abnormalities and Hippocampal BDNF Downregulation

Regarding emotional behavior, simulated weightlessness led to decreased sucrose preference and prolonged feeding latency in the novelty-suppressed feeding test in rats, suggesting the emergence of anxiety/depression-like behaviors. This is consistent with previous studies reporting that simulated weightlessness can induce emotional and cognitive changes. This study found a significant decrease in hippocampal BDNF levels, providing a molecular basis for weightlessness-induced emotional disorders. BDNF is a key neurotrophic factor involved in neuronal survival, synaptic plasticity, and learning and memory, and its downregulation is closely related to the pathogenesis of depression and anxiety. Previous studies have shown that simulated weightlessness can inhibit the hippocampal BDNF-TrkB pathway in mice, leading to cognitive dysfunction [7]. The results of this study are consistent with this and further suggest that domperidone, while improving gastrointestinal motility, can significantly elevate hippocampal BDNF levels. This may be related to its regulation of brain–gut axis function and indirect influence on the central neurotrophic state. This finding expands the understanding of the mechanism of action of prokinetic drugs, suggesting that they may have multi-target regulatory effects beyond the gastrointestinal tract.

### 3.3. Impaired Gut Homeostasis: Multiple Pathological Bases of Weightlessness-Induced FD

The impact of weightlessness on the digestive system is complex and multifaceted. In addition to directly affecting smooth muscle contraction and neuroendocrine function, studies have confirmed that simulated weightlessness can comprehensively impair gut homeostasis. A recent study published in BMC Microbiology using a hindlimb suspension rat model found that long-term microgravity exposure significantly increased hepatic lipid deposition, oxidative stress, and inflammatory responses. It also increased the proportion of opportunistic pathogens in the gut, impaired barrier function, and disrupted the metabolism of secondary bile acids (ursodeoxycholic acid, lithocholic acid) and tryptophan [22]. That study confirmed that the gut microbiota is a key target for maintaining gut–liver axis homeostasis, providing a new perspective for understanding digestive system dysfunction in weightlessness environments.

Simulated weightlessness not only damages the intestinal mucosal barrier, manifested by disrupted intestinal villus structure and loss of tight junction integrity between epithelial cells, leading to increased intestinal permeability [23], but also significantly perturbs the composition and spatial distribution of the gut microbiota [24]. For example, it alters key community structures such as the Firmicutes/Bacteroidetes ratio, accompanied by a reduction in beneficial metabolites (e.g., short-chain fatty acids) [25]. These changes are often accompanied by intestinal immune imbalance. One of the core mechanisms involves the activation of classical inflammatory signaling pathways such as Toll-like receptor 4 (TLR4)/myeloid differentiation primary response 88 (MyD88)/nuclear factor kappa-B (NF-κB), which promotes the expression of pro-inflammatory cytokines (e.g., TNF-α, IL-6), thereby exacerbating the low-grade inflammatory state of the gut [26].

The damaged intestinal barrier, disturbed microbiota and their metabolites, and persistent immune-inflammatory responses can all act as potent signals affecting the central nervous system via pathways such as the vagus nerve and blood circulation. Through the bidirectional communication of the brain–gut axis, they participate in the development and progression of multidimensional symptoms in FD, including anorexia, dysmotility, and emotional abnormalities [27].

### 3.4. Multi-Target Regulatory Effects of Domperidone and Clinical Implications

Domperidone treatment effectively improved gastrointestinal motility and behavioral abnormalities in rats and partially corrected the brain–gut peptide disorder [26]. While its prokinetic effect is well established, the present study shows that these improvements are associated with a partial reversal of the imbalanced brain–gut peptide profile. However, because this study did not include a domperidone-only control group (i.e., domperidone administered to non-suspended rats), the possibility of nonspecific drug effects on motility or behavior cannot be excluded.

Domperidone is a peripheral dopamine D2 receptor antagonist with limited blood–brain barrier penetration. Therefore, its observed effects on brain–gut peptide expression, particularly the upregulation of ghrelin and GHSR-1a mRNA in the hypothalamus, are likely indirect. Potential indirect pathways include the following: (1) improvement in nutritional status secondary to enhanced gastric emptying; (2) reduction in visceral discomfort or bloating, which may alter vagal afferent signaling; or (3) modulation of enteric nervous system activity that subsequently influences central neuropeptide expression via gut-brain circuits. Direct central action of domperidone, while theoretically possible at high doses or after blood–brain barrier disruption, remains speculative without direct evidence such as cerebrospinal fluid drug concentration measurement or central receptor occupancy studies.

The finding that domperidone treatment was associated with elevated hypothalamic ghrelin and GHSR-1a mRNA, along with reduced VIP and CCK expression, provides a correlative link between drug treatment and brain–gut axis changes. Nevertheless, causality cannot be inferred from the current data. Future studies using brain-permeable D2 antagonists, conditional receptor knockouts, or selective vagal deafferentation are needed to determine whether central or peripheral mechanisms predominate.

From a translational perspective, this study demonstrates that the tail-suspension model can be used to evaluate candidate interventions for spaceflight-associated digestive dysfunction in a standardized and reproducible manner. However, given the multifactorial pathogenesis of FD-like changes under simulated weightlessness—including brain–gut peptide imbalance, potential intestinal barrier alterations, microbiota dysbiosis, and immune activation—single-target interventions such as domperidone alone may not fully reverse all pathological features. Future strategies might explore multi-target combinations, such as prokinetics paired with probiotics, intestinal barrier protectants, or selective brain–gut peptide receptor modulators.

### 3.5. Limitations and Future Directions

The tail-suspension model is a well-established method for simulating microgravity, but it is also a significant physical and psychological stressor. Many of the observed changes could be partially attributable to chronic stress rather than microgravity per se. Future studies should include stress-only control groups to dissociate these effects. This study focused on changes in the circulating brain–gut peptide profile, providing direct evidence for the neuroendocrine mechanism of weightlessness-induced FD. However, certain limitations exist: First, this study did not achieve dynamic monitoring of the time course of brain–gut peptide changes, so the causal temporal relationship between brain–gut peptide imbalance and behavioral changes cannot be determined. Second, sequencing analysis of gut microbiota composition and function was not performed, preventing assessment of the contribution of microbiota–brain–gut axis interactions. Third, no domperidone-only control group was included, so nonspecific drug effects cannot be excluded. Fourth, behavioral interpretations are potentially confounded by changes in appetite and abdominal discomfort, as noted above. Finally, the mechanism of action of domperidone has not been causally validated through means such as receptor antagonism or gene knockout.

Future research needs to further integrate multi-dimensional data, including the expression of tight junction proteins in intestinal mucosal tissue, gut metagenomics/metabolomics, and local immune indicators such as mesenteric lymph nodes. At the same time, using specific agonists or antagonists targeting key brain–gut peptide receptors (e.g., ghrelin receptor, VIP receptor) or inflammatory pathways (e.g., NF-κB inhibitors) for intervention will help to elucidate, at a causal level, the specific temporal sequence and weight of each factor in the “weightlessness–gut homeostasis disturbance–brain–gut axis dysregulation–FD” pathway. Furthermore, combining neuroimaging techniques (e.g., fMRI) to observe functional changes in the limbic system under weightlessness will provide more direct evidence for the brain–gut axis mechanism. From a model standardization and translational perspective, future efforts should prioritize cross-laboratory validation of the tail-suspension model, harmonization of outcome measures, and integration with complementary tools such as organoid systems or humanized microbiota models to enhance mechanistic insight and translational relevance. Based on these in-depth studies, it is expected that multi-target digestive function protection strategies for the special space environment can be developed, laying the foundation for safeguarding the health of astronauts during long-term deep space exploration missions.

## 4. Materials and Methods

### 4.1. Animals and Ethical Approval

All animal experiments were conducted in accordance with the guidelines of the Institutional Animal Care and Use Committee. Thirty healthy male Wistar rats (6–8 weeks old, weighing 280–320 g) were purchased from Beijing Vital River Laboratory Animal Technology Co., Ltd. (Beijing, China) (license number: SCXK (Jing) 2021-0006; animal certificate number: NO.110324241106764912). The rats were housed under specific pathogen free (SPF) conditions at the Laboratory Animal Facility of China Agricultural University (license number: SYXK (Jing) 2021-0012), with controlled temperature (22–25 °C), humidity (50–60%), and a 12 h light/dark cycle. Animals had free access to standard chow and water ad libitum. All procedures were approved by the Animal Ethics Committee of China Agricultural University. After one week of acclimatization, the rats were randomly assigned to experimental groups.

### 4.2. Simulated Microgravity Model and Drug Administration

The simulated weightlessness model was established using the standard tail-suspension method. Briefly, rats in the model and treatment groups were suspended individually by the tail at a 30° head-down tilt angle for 21 consecutive days, allowing free movement with forelimb support while the hindlimbs were non-weight-bearing. The suspension apparatus was checked daily to ensure proper positioning and adequate tail circulation. Control rats were housed under identical conditions without suspension.

Thirty rats were randomly divided into three groups (n = 10 per group): (1) control group: received normal saline (2 mL/100 g body weight) by daily oral gavage for 21 days without suspension; (2) HU (hindlimb unloading) group: subjected to 21-day tail suspension and received normal saline (2 mL/100 g) daily by oral gavage; (3) domperidone group: subjected to 21-day tail suspension and received domperidone solution (0.27 mg/mL, 2 mL/100 g body weight, equivalent to clinical dose) daily by oral gavage. Domperidone tablets (10 mg/tablet, batch number: H10910003, Xi’an Janssen Pharmaceutical Ltd., Xi’an, China) were ground into fine powder and dissolved in 0.9% saline to prepare the solution fresh daily.

### 4.3. General Condition Monitoring

Throughout the 21-day experimental period, body weight and daily food intake were recorded weekly. General observations including fur condition, fecal characteristics, mental state, and locomotor activity were documented.

### 4.4. Behavioral Assessments

#### 4.4.1. Sucrose Preference Test (SPT)

The sucrose preference test was performed on day 20 to evaluate anhedonia-like behavior. The test consisted of a 48 h training period, 24 h food and water deprivation, and a 3 h test period. During training, rats were exposed to two bottles of 1% sucrose solution for 24 h, followed by one bottle of 1% sucrose and one bottle of water for another 24 h, with bottle positions switched every 12 h to avoid side preference. After 24 h of deprivation, rats were given free access to two pre-weighed bottles containing 1% sucrose solution and tap water for 3 h. Sucrose preference (%) was calculated as [sucrose intake (g)/(sucrose intake (g) + water intake (g))] × 100%.

#### 4.4.2. Novelty-Suppressed Feeding Test (NFT)

The novelty-suppressed feeding test was conducted on day 21 to assess anxiety-like behavior. Rats were fasted for 24 h prior to testing. The test apparatus consisted of an open field arena (50 × 50 × 40 cm) with three food pellets placed in the center. Each rat was placed in a corner of the arena, and the latency to begin feeding (defined as the time to first bite of food) was recorded within a 15 min observation period. Rats that did not feed within 15 min were assigned a latency of 15 min.

All behavioral tests were performed by experimenters blinded to group assignment.

### 4.5. Gastrointestinal Motility Measurement

Following behavioral tests, rats were fasted for 24 h with free access to water. On the day of sacrifice, each rat received an intragastric administration of black semi-solid nutritional paste (1 mL/100 g body weight) containing 5% carboxymethylcellulose sodium, 8% milk powder, 4% sucrose, 4% starch, and 2% activated carbon. After 30 min, rats were deeply anesthetized with 10% chloral hydrate (1 mL/100 g, intraperitoneal injection). The abdomen was opened, and the stomach and small intestine were carefully removed.

For gastric emptying measurement, the stomach was weighed (total weight, M1), opened along the greater curvature, rinsed with saline to remove contents, blotted dry, and reweighed (net weight, M2). Gastric emptying rate (%) was calculated as [1 − (M1 − M2)/weight of paste administered] × 100%.

For intestinal transit measurement, the entire small intestine from pylorus to cecum was gently stretched on a wet surface. The total intestinal length (L) and the distance traveled by the charcoal front (L1) were measured. Intestinal propulsion rate (%) was calculated as (L1/L) × 100%.

### 4.6. Sample Collection

Blood samples were collected from the abdominal aorta and allowed to clot at room temperature for 30 min, followed by centrifugation at 3000 rpm for 15 min at 4 °C. Serum was aliquoted and stored at −80 °C until analysis. The stomach, hypothalamus, colon, and hippocampus were rapidly dissected on ice. Gastric tissue was divided: fixed in 4% paraformaldehyde for histopathological examination, and the remaining portions of stomach, hypothalamus, colon, and hippocampus were snap-frozen in liquid nitrogen and stored at −80 °C for subsequent molecular analyses.

### 4.7. Enzyme-Linked Immunosorbent Assay (ELISA)

Serum levels of brain–gut peptides, including vasoactive intestinal peptide (VIP), cholecystokinin (CCK), substance P (SP), gastrin (GAS), motilin (MTL), calcitonin gene-related peptide (CGRP), ghrelin, and nesfatin-1, were measured using commercial ELISA kits (Beijing Jinzhiyan Biotechnology Co., Ltd., Beijing, China) according to the manufacturer’s instructions. Colon tissue was homogenized in ice-cold phosphate-buffered saline (PBS) and centrifuged at 3000 rpm for 20 min at 4 °C, and the supernatant was collected for 5-hydroxytryptamine (5-HT) measurement. Hippocampal tissue was processed similarly for brain-derived neurotrophic factor (BDNF) measurement. Protein concentrations were normalized to total protein content determined by BCA assay. All assays were performed in duplicate, and the intra- and interassay coefficients of variation were below 10%.

### 4.8. Quantitative Real-Time PCR (qRT-PCR)

Total RNA was extracted from gastric and hypothalamic tissues using an RNA extraction kit (Aidlab Biotechnologies, Beijing, China; cat. no. RN28) following the manufacturer’s protocol. RNA purity and concentration were assessed by spectrophotometry (A260/A280 ratio between 1.8 and 2.0). Complementary DNA (cDNA) was synthesized from 1 μg of total RNA using a reverse transcription kit (Promega, Madison, WI, USA; cat. no. A5000).

Quantitative real-time PCR was performed on an Abbott m2000rt real-time PCR system using SYBR Green Master Mix (Promega). The PCR conditions were as follows: initial denaturation at 95 °C for 1 min, followed by 40 cycles of 95 °C for 20 s and 60 °C for 1 min. The primer sequences for target genes (VIP, CCK, CGRP, SP, GAS, ghrelin, GHSR-1a, and GAPDH as internal control) were designed and validated in previous studies. Relative gene expression was calculated using the 2^(−ΔΔCt)^ method. All reactions were performed in triplicate.

The primer sequences used for qRT-PCR are listed in Supplementary Table S1. All primers were validated for amplification efficiency and specificity prior to use.

### 4.9. Histopathological Examination

Gastric tissue fixed in 4% paraformaldehyde for 24 h was dehydrated through graded ethanol, cleared in xylene, and embedded in paraffin. Sections of 5 μm thickness were cut and stained with hematoxylin and eosin (H&E). Histopathological evaluation was performed under a light microscope (Olympus, Tokyo, Japan) by a pathologist blinded to the experimental groups. Gastric mucosal integrity, epithelial cell arrangement, glandular structure, and presence of inflammatory infiltration, erosion, or ulceration were assessed.

### 4.10. Statistical Analysis

All data were expressed as mean ± standard deviation (SD). Statistical analyses were performed using GraphPad Prism version 9.4.1 (GraphPad Software, San Diego, CA, USA). Comparisons among multiple groups were conducted using one-way analysis of variance (ANOVA) followed by Tukey’s honestly significant difference (HSD) post hoc test for pairwise comparisons. A value of *p* < 0.05 was considered statistically significant.

### 4.11. Data Availability

All relevant data generated during this study are included in this published article. The datasets used and/or analyzed during the current study are available from the corresponding author upon reasonable request.

### 4.12. Use of Generative Artificial Intelligence

During the preparation of this manuscript, the authors used generative artificial intelligence (GenAI) tools (specifically, a large language model) to assist with language refinement and text formatting to improve readability. The authors reviewed and edited all AI-generated content and take full responsibility for the final version of the manuscript. GenAI was not used for study design, data collection, analysis, interpretation, or figure generation.

## 5. Conclusions

The 21-day tail-suspension model recapitulates key FD-like changes in rats—gastrointestinal dysmotility, altered behavioral responses, and brain–gut peptide imbalance—without organic gastric pathology. Domperidone treatment improves these outcomes and partially reverses peptide imbalances. However, the model involves chronic stress, behavioral changes may reflect appetite effects, causality is not established, and domperidone’s central effects are likely indirect. These findings provide correlative evidence for brain–gut axis involvement in simulated microgravity-induced FD and support the tail-suspension model as a preclinical platform for evaluating therapeutic interventions. Future studies should include stress-only controls, assess inflammatory/barrier markers, and use targeted receptor antagonists or genetic models to establish causality.

## Figures and Tables

**Table 1:** Body weight changes over 21 days in control, HU, and domperidone groups.

Group	1 Day (g)	7 Day (g)	14 Day (g)	21 Day (g)
Control	296.7 ± 7.6	316.3 ± 9.8	325.3 ± 4.4	334.2 ± 5.1
HU	295.4 ± 5.4	301.8 ± 8.8 *	318.2 ± 2.9 *	324.7 ± 3.8 *
Domperidone	295.8 ± 6.2	309.5 ± 6.4	322.1 ± 4.6 #	330.5 ± 5.2 #

Note: * *p* < 0.05 vs. Control; # *p* < 0.05 vs. HU. Control: control group; HU: simulated weightlessness model group; domperidone: domperidone intervention group.

## Data Availability

The data presented in this study are available on request from the corresponding author due to privacy/ethical restrictions.

## Supplementary Materials

The following supporting information can be downloaded at: https://www.mdpi.com/article/10.3390/ijms27114915/s1.

## Abbreviations

5-HT	5-Hydroxytryptamine (Serotonin)
BDNF	Brain-Derived Neurotrophic Factor
CCK	Cholecystokinin
CGRP	Calcitonin Gene-Related Peptide
cDNA	Complementary DNA
D2	Dopamine Receptor D2
ELISA	Enzyme-Linked Immunosorbent Assay
FD	Functional Dyspepsia
fMRI	Functional Magnetic Resonance Imaging
GAPDH	Glyceraldehyde-3-Phosphate Dehydrogenase
GAS	Gastrin
GHSR-1a	Growth Hormone Secretagogue Receptor 1a (Ghrelin Receptor)
GIP	Gastric Inhibitory Polypeptide/Glucose-Dependent Insulinotropic Polypeptide
GLP-1	Glucagon-Like Peptide-1
HE	Hematoxylin and Eosin
HU	Hindlimb Unloading (Simulated Weightlessness Model Group)
IL-6	Interleukin-6
MTL	Motilin
MyD88	Myeloid Differentiation Primary Response 88
Nesfatin-1	Nesfatin-1 (NEFA/Nucleobindin2-Encoded Protein)
NF-κB	Nuclear Factor kappa-B
NFT	Novelty-Suppressed Feeding Test
PBS	Phosphate-Buffered Saline
PCR	Polymerase Chain Reaction
qRT-PCR	Quantitative Real-Time Polymerase Chain Reaction
SD	Standard Deviation
SP	Substance P
SPF	Specific Pathogen-Free
SPT	Sucrose Preference Test
TLR4	Toll-Like Receptor 4
TNF-α	Tumor Necrosis Factor-alpha
VIP	Vasoactive Intestinal Peptide
