# Supplementary material for: Tail-Suspension Model of Simulated Microgravity-Induced Functional Dyspepsia in Rats: Behavioral, Motility, and Brain–Gut Peptide Alterations
Source: Int J Mol Sci. 2026 May 29;27(11):4915. doi: 10.3390/ijms27114915 (PMC13256725; doi:10.3390/ijms27114915)
Supplement: Supplementary file 1 [file ijms-27-04915-s001.zip › ijms-4261090-supplementary.pdf]

Table S1 primers sequences

Primer name	Forward primer（5’-3’）	Reverse primer（5’-3’）
VIP	GCCGGTCAAACGACACTCTG	CCTCACTGCTCCTCTTCCCATT
CCK	CAGGTCCGCAAAGCTCCC	CCGAAATCCATCCAGCCCAT
SP	CGGTGCCAACGATGATCTAA	GTAGTTCTGCATTGCGCTTC
GAS	AGATGCCTCGACTGTGTGTG	CATTGGTGGCCTCTGTTTCT
CGRP	CCTTTCCTGGTTGTCAGCA	GCTCCCTGACTTTCATCTGC
Ghrelin	CAGGAGCTCAGTACCAGCAG	GAGGCAGAAGCTGGATGTGA
GHSR-1a	ACCACCACCAACCTCTAC	CAGCTCTCGCTGACAAAC
GAPDH	GACATGCCGCCTGGAGAAAC	AGCCCAGGATGCCCTTTAGT
